# Antiproliferative Copper(II) Complexes Bearing Mixed Chelating Ligands: Structural Characterization, ROS Scavenging, In Silico Studies, and Anti-Melanoma Activity

**DOI:** 10.3390/pharmaceutics14081692

**Published:** 2022-08-14

**Authors:** Rodica Olar, Catalin Maxim, Mihaela Badea, Mihaela Bacalum, Mina Raileanu, Speranta Avram, Nataša Čelan Korošin, Teodora Burlanescu, Arpad Mihai Rostas

**Affiliations:** 1 Faculty of Chemistry, Department of Inorganic Chemistry, University of Bucharest, 90-92 Panduri Str., 050663 Bucharest, Romania; 2 Horia Hulubei National Institute for Physics and Nuclear Engineering, Department of Life and Environmental Physics, 30 Reactorului Str., 077125 Magurele-Ilfov, Romania; 3 Faculty of Physics, Department of Electricity, Solid State and Biophysics, University of Bucharest, 405A Atomiștilor Str., 077125 Magurele-Ilfov, Romania; 4 Faculty of Biology, Department of Anatomy, Animal Physiology and Biophysics, University of Bucharest, 91-95, Splaiul Independenței, 050095 Bucharest, Romania; 5 Faculty of Chemistry and Chemical Technology, University of Ljubljana, Večna pot 113, 1000 Ljubljana, Slovenia; 6 Laboratory of Optical Processes in Nanostructure Materials, National Institute of Materials Physics, 405A Atomiștilor Str., 077125 Magurele-Ilfov, Romania; 7 Laboratory of Atomic Structures and Defects in Advanced Materials, LASDAM, National Institute of Materials Physics, 405A Atomiștilor Str., 077125 Magurele-Ilfov, Romania

**Keywords:** Cu(II) complex, structure, ROS, cytotoxicity, melanoma cells, bioinformatics, ADME-Tox

## Abstract

Melanoma is a skin cancer characterized by rapid growth and spread for which current therapies produce both resistance and increased risk of infection. To develop new anti-melanoma biocompatible species, the series of complexes Cu(N-N)(bzac)(X)⋅nH_2_O (N-N: 1,10-phenanthroline/2,2′-bipyridine, Hbzac: 1-phenyl-1,3-butanedione, X: NO_3_/ClO_4_, and *n* = 0, 1) was studied. Single-crystal X-ray diffraction revealed a mononuclear structure for all complexes. The ability of the complexes to scavenge or trap reactive oxygen species such as O_2_⋅^−^ and HO⋅ was proved by EPR spectroscopy experiments. All complexes inhibited B16 murine melanoma cells in a dose-dependent and nanomolar range, but the complexes with 1,10-phenanthroline were more active. Moreover, comparative activity on B16 and healthy BJ cells revealed a therapeutic index of 1.27–2.24. Bioinformatic methods were used to calculate the drug-likeness, pharmacokinetic, pharmacogenomic, and pharmacodynamic profiles of the compounds. The results showed that all compounds exhibit drug-likeness features, as well as promising absorption, distribution, metabolism, and excretion (ADME) properties, and no toxicity. The pharmacodynamics results showed that the neutral species appear to be good candidates for antitumor molecular targets (Tyrosyl-DNA phosphodiesterase 1, DNA-(apurinic or apyrimidinic site) lyase or Kruppel-like factor 5). Furthermore, the pharmacogenomic results showed a good affinity of the copper(II) complexes for the human cytochrome. These results recommend complexes bearing 1,10-phenanthroline as good candidates for developing drugs to melanoma alternative treatment.

## 1. Introduction

Cancer is a difficult disease to treat because both organic and inorganic drugs must have low toxicity and reduced side effects besides efficacy. Among various types of cancers, melanoma is less common than other types of skin cancer, such as keratinocyte carcinomas, but it is more likely to grow and spread [1]. The main environmental risk factor for melanoma development is UV light exposure, which causes genetic mutations in melanocytes [2].

Chemotherapeutic treatment of melanoma includes dacarbazine as a single agent and carboplatin in combination with paclitaxel as combinatory drug therapy [1,3]. These therapies lead to severe side effects, including acquired resistance along with an increased risk of infection. Consequently, challenges and opportunities remain around developing new chemotherapeutics species for melanoma treatment that can overcome the observed deficiencies of current drugs.

Hence, in the constant search for new drugs with lower toxicity and reduced side effects, Cu(II) complexes are preferred, considering their reduced systemic toxicity due to the essential character of this ion [4,5,6,7,8]. Thus, several Cu(II) species with multidentate ligands have been designed as non-toxic species for healthy tissues, but with high activity and selectivity in melanoma cells.

The first Cu(II) complex with such activity arose from the observation that the mechanism of action of elesclomol (esl), a compound active in metastatic melanoma, comprises its complexation with Cu(II) in both extracellular and intracellular fluids. Therefore, the [Cu(esl)]^2+^ species is considered to be the active form, and assays against MDA-MB435 melanoma cells yielded a value of half-maximal inhibitory concentration (IC_50_) of less than 100 nM. Thus, the indirect inhibitory effect of esl is related to Cu(II)-mediated oxidative stress in tumor cells by producing a high level of hydrogen peroxide [9].

Several complexes with nitrogen-based heterocyclic ligands with chelating abilities were found to be good candidates for the treatment of melanoma based on in vitro assays. Among them, the species [Cu(phen)(hpo)]NO_3_ (phen: 1,10-phenanthroline; Hhpo: (E)-1-(2′-hydroxyphenyl)-3-phenylprop-2-en-1-one derivatives) exhibits better activity compared to cisplatin against G361 melanoma cell lines [10], while the binuclear complexes [Cu_2_phen_2_Cl_2_(μ^2^-X)_2_] (X: Cl [11]; N_3_ [12]) proved activity against B16 melanoma, with both acting in the micromolar range. Some complexes such as [Cu_2_(terph)phen_4_](terph) (H_2_terph: terephthalic acid), [Cu_2_(oda)phen_4_](ClO_4_)_2_ (oda: octane-1,8-dioic acid) [13], and [Cuphen(dmgly)(OH_2_)](NO_3_) (Hdmgly: dimethylglycine) [14] showed activity against a range of melanoma cell lines in the micromolar level, with remarkable selectivity for SK-MEL-5 cells.

Moreover, the heteroleptic species [Cu(phen)(smtr/sdmx)^2^] (Hsmtr: sulfameter; Hsdmx: sulfadimethoxine) were found to be active against the UACC-62 melanoma cells where their activity was associated with their efficient metallonuclease activity [15].

With similar nuclease-like activity the complexes [Cu(phen)_2_(OH_2_)](ClO_4_)_2_ [16], [Cu(bpy/phen)_2_(pmtp)](ClO_4_)_2_ (bpy: 2,2′-bipyridine; pmtp: 5-phenyl-7-methyl-1,2,4-triazolo[1,5-a]pyrimidine) [17] and [Cu(bpy/phen)(dmtp)_2_(OH_2_)](ClO_4_)_2_·dmtp (dmtp: 5,7-dimethyl-1,2,4-triazolo[1,5-a]pyrimidine) were reported [18]. All complexes were active in the micromolar range against B16 murine melanoma cells and showed low toxicity against healthy BJ cells. Still, it is worth noting that [Cu(bpy)(dmtp)_2_(OH_2_)](ClO_4_)_2_·dmtp showed better cell selectivity with a therapeutic index of 12.82 at 48 h [18].

Other Cu(II) compounds active in the micromolar range on B16 melanoma cell lines have been developed based on pyrazole [19], pyridine [20,21,22], thiophenecarboxylato [23] derivatives, or even doxorubicin, a well-known organic-based antitumor drug [24].

Herein, we report the antineoplastic activity of some already known copper (II) complexes, [Cu(phen)(bzac)(ClO_4_)] (**1**), [Cu(bpy)(bzac)]ClO_4_ (**3**) [25], [Cu(bpy)(bzac)(NO_3_)] (**4**) [26] and, for a new one, [Cu(phen)(bzac)(NO_3_)]⋅H_2_O (**2**) (Hbzac: 1-phenyl-1,3-butanedione). The biological activity of the complexes was evaluated on B16 murine melanoma cells. We also addressed the effects of the chemical structures of these species on their anti-tumor activities.

In addition, bioinformatic tools [27] were used to predict the drug-like profile, absorption, distribution, metabolism, and elimination (ADME) profiles of the studied compounds [28], with an emphasis on predicting the affinities of molecular targets. Based on our experience in the bioinformatics of new drug candidates [29], we aimed to evaluate the Cu(II) complexes interactions with human antitumor targets.

## 2. Materials and Methods

### 2.1. Materials and Physical Measurements

All the chemicals that were used were purchased from Sigma-Aldrich (St. Louis, MO, USA) (copper(II) nitrate hexahydrate (≥99.99% trace metals basis), 1,10-phenanthroline (phen, 99%) 2,2′-bipyridine (bpy, 99%) and 1-phenyl-1,3-butanedione (Hbzac, 99%)) in reagent grade, and were used as received without further purification.

The C, N, and H chemical analyses were carried out with a EuroEA elemental analyzer. Fourier Transform Infrared spectroscopy (FTIR) spectra were recorded with a Tensor 37 spectrometer (Bruker, Billerica, MA, USA) in the 400–4000 cm^−1^ range. A Jasco V 670 spectrophotometer (Jasco, Easton, MD, USA) with Spectralon as a standard in the 200–1500 nm range was used to record the UV-Vis spectra in solid-state. A continuous wave X-Band Bruker EMX plus EPR spectrometer (Bruker, Karlsruhe, Germany) equipped with a Bruker X-SHQ 4119HS-W1 X-Band resonator was used for the X-band EPR measurements. The parameters for the X-Band measurements were set as follows: microwave frequency 9.879 GHz, microwave power 6.325 mW, modulation amplitude 0.2 mT, conversion time 74.17 ms, and time constant 40.96 ms at 35 scans. All measurements were carried out at room temperature. Simultaneous Thermogravimetric/Differential Scanning Calorimetry–Mass spectroscopy (TG-DSC/MS) measurements were performed on a TGA/DSC1 Thermal Analyser (Mettler Toledo, Schwarzenbach, Switzerland), coupled to a Vacuum Thermostar Mass Spectrometer (Pfeiffer, Asslar, Germany) under the following conditions: isothermal conditioning at 25 °C, temperature range from 25 to 800 °C, heating rate 5 °C/min, dynamic air atmosphere with a flow rate of 100 mL/min, sample masses of 2–4 mg, 150 μL platinum crucible; empty crucible served as reference. The blank curve was subtracted. Evolved gasses were introduced into the Mass Spectrometer via a 75 cm heated capillary.

### 2.2. Synthesis of Complexes

The complexes (**1**), (**3**), and (**4**) were synthesized as reported [25,26].

The complex [Cu(bzac)(phen)NO_3_]⋅H_2_O (**2**) was prepared as follows: to a solution containing copper(II) nitrate hexahydrate (0.244 g, 1 mmol) in 25 mL ethanol, a solution containing phen (0.180 g, 1 mmol) was added in 10 mL ethanol. The reaction mixture was magnetically stirred at 50 °C for 1 h, and then a solution obtained by dissolving Hbzac (0.162 g, 1 mmol) in 10 mL ethanol was added. The dark green single crystals obtained after the solution stood at room temperature for several days were isolated. The resulting solution was further concentrated, and the obtained microcrystals were filtered off, washed several times with cold ethanol, and dried in air. Yield 86%. Anal. Calcd. for C_22_H_18_CuN_3_O_6_ (%): C, 54.60; H, 3.75; N, 8.68; Found (%): C, 54.67; H, 3.68; N, 8.71. 

### 2.3. Crystal Structural Analysis

The structure was solved by direct methods and refined using full-matrix least-squares techniques based on F2. The non-H atoms were refined using anisotropic displacement parameters. The calculations were performed using the crystallographic software package SHELX-2018 [30]. The crystallographic data (excluding structure factors) have been deposited with the Cambridge Crystallographic Data Centre with CCDC reference number 2184808. These data can be obtained free of charge from http://www.ccdc.cam.ac.uk/conts/retrieving.html (accessed on 28 June 2022), or the Cambridge Crystallographic Data Centre, 12 Union Road, Cambridge CB2 1EZ, UK; fax: (+44) 1223-336-033; or e-mail: deposit@ccdc.cam.ac.uk.

### 2.4. In Vitro Cytotoxicity Assay

#### 2.4.1. Cell Culture Conditions

Human fibroblast cells (BJ-ATCC CRL-2522, USA) were grown in minimal essential medium (MEM) supplemented with 2 mM L-Glutamine, 10% fetal calf serum (FCS), 100 units/mL of penicillin, and 100 µg/mL of streptomycin at 37 °C in a humidified incubator under an atmosphere containing 5% CO_2_. Mouse melanoma cells (B16-ATCC CRL-6475, USA) were grown in DMEM (Dulbecco’s Modified Eagle Medium) supplemented with reagents like MEM. All cell cultivation media and reagents were purchased from Biochrom AG (Berlin, Germany) and Sigma-Aldrich (Darmstadt, Germany).

#### 2.4.2. Cell Viability Assay

Cell viability was evaluated using the MTT assay (3-(4,5-dimethylthiazol-2-yl)-2,5-diphenyltetrazolium bromide) as previously described [14]. Briefly, BJ and B16 cells were seeded in 96-well plates at densities of 10,000 cells/well and 7000 cells/well and further cultured in the medium for 24 h. The next day, the investigated compounds were added at a concentration of 1 to 25 μM for 24 and 48 h. The cells cultivated in medium without the studied samples served as negative controls. After incubation for the desired times, the medium was replaced with MTT solution (1 mg/mL) and incubated for an additional 4 h at 37 °C. Finally, the medium was collected, and DMSO was used to dissolve the insoluble formazan product. A Mithras 940 (Berthold, Bad Wildbad, Germany) plate reader was used to measure the absorbance of the samples at 570 nm. The data were corrected for background, and the percentage of viable cells was obtained using the following equation:

Cell viability = [(A_570_ of treated cells)/(A_570_ of untreated cells)] × 100 (%)

Half-maximal inhibitory concentration (IC_50_) was determined by fitting the data with a sigmoidal logistic function using Origin 8.1 (Microcal Inc., Northampton, MA, USA) software.

#### 2.4.3. Cell Cycle Analysis

Twenty-four hours after treatment of B16 cells with concentrations of 1 and 5 μM of each compound, cells were harvested and fixed with pre-chilled 70% ethanol solution at −20 °C. Cells were incubated with 0.2 mg/mL RNAse and 20 μg/mL propidium iodide (PI) in a 0.1% Triton TX-100 solution, in the dark, for 30 min at 37 °C. Cell cycle distribution was analyzed using a Beckman Coulter Cell Lab Quanta SC Flow Cytometer (Indianapolis, IN, USA), and the data were analyzed using the Quanta Analysis software.

### 2.5. Computational Strategy

#### 2.5.1. Molecular Modeling of Complexes

Starting from the 3D structures of the compounds downloaded as pdb files in the Molecular Operating Environment (MOE) software [31], the Simplified Molecular Input Line Entry (SMILES) file of the compounds for further bioinformatics and cheminformatics analysis was obtained. In addition, the optimized energies of the compounds were determined using the MOE software. The energies of the structures were minimized using Forcefield MMFF94x with a gradient of 0.05. After minimization, the Gasteiger partial charges were applied to all compounds.

#### 2.5.2. Assessment of Compounds’ Drug- and Lead-Likeness Features

To evaluate the features of drug- and lead-likeness, the complexes were subjected to Lipinski [32], Ghose [33], Veber [34], and Egan [35] filters using the SwissADME web service [36].

Under these rules, bioactive drugs are not expected to violate more than one of the criteria: (i) Lipinski filter—The molecular weight should not exceed 500 g/mol, the partition coefficient between octanol and water (Log P(*o*/*w*)) should not exceed 5, the number of hydrogen bond acceptors should not exceed 10, and the number of hydrogen bond donors should not exceed 5; (ii) Ghose filter—The molecular weight should be between 160 and 480 g/mol, the Log P(*o*/*w*) should be between −0.4 and 5.6, the molar refractivity should be between 40 and 130 m^3^/mol, and the number of atoms should be between 20 and 70; (iii) Veber rule—the number of rotatable bonds should not be more than 10 and the total polar surface area should not be greater than 140 Ǻ^2^, and (iv) Egan filter—The Log P(*o*/*w*) should not be more than 5.88 and the total polar surface area should not be greater than 131 Ǻ^2^.

#### 2.5.3. Computational Pharmacokinetics and Pharmacogenomics Profiles of Copper(II) Complexes

The SMILES files of copper(II) complexes were used to predict their absorption, distribution, excretion, and toxicity (ADE-Tox) profiles using the pkCSM database [37]. The following criteria were selected for our research: (i) intestinal absorption (percentage)—A molecule with an absorption rate of less than 30% is considered poorly absorbed; (ii) blood–brain barrier (BBB) permeability represented as log BBB (logarithm of brain-to-plasma drug concentration ratio)—A value greater than 0.3 indicates high BBB permeability, while a value less than 1 indicates low BBB permeability and (iii) substrate of renal organic cation transporter 2 (OCT2), the primary renal uptake transporter expressed on the basolateral side of the proximal tubule. The predicted toxic profile of the compounds was a significant aspect of our research. We assessed AMES toxicity, hepatotoxicity, cardiotoxicity, and median lethal dose (LD_50_).

Considering the Pharmacogenomics Profiles of molecular complexes, we investigated the potential of the compounds to serve as inhibitors or substrates for cytochromes involved in the metabolism of many antitumor drugs, such as CYP2D6, CYP3A4, CYP1A2, CYP2C19, and CYP2C9, to predict their pharmacogenomic profile.

#### 2.5.4. Compounds’ Computational Pharmacodynamic Profiles

The pharmacodynamic features and possible molecular mechanisms by which copper(II) complexes modulate human enzymes involved in cancer are less represented in bioinformatics and pharmaceutical databases. In this context, the SuperPred database [38] was used to establish the probability of binding copper(II) complexes to enzymes involved in antitumor treatments based on logistic regression machine learning models. The binding data and resulting model accuracy are based on 646 human targets. In addition, therapeutic indications of the predicted targets [39] were identified.

## 3. Results and Discussion

**Figure 1:**
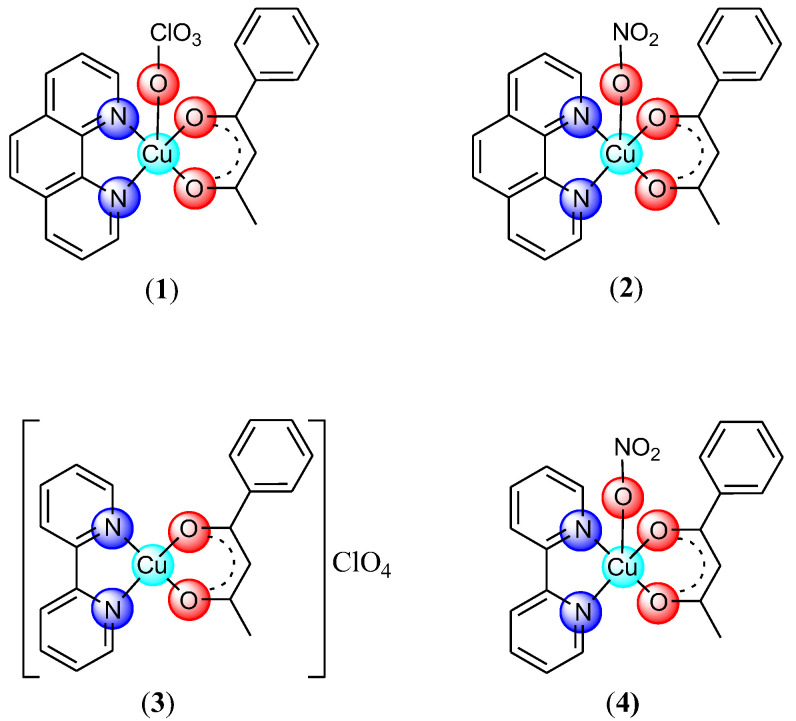
The structure of complexes.

The stepwise reaction of copper perchlorate or nitrate with 1,10-phenanthroline (phen)/2,2′-bipyridine (bpy) and 1-phenyl-1,3-butanedione (Hbzac) in a molar ratio of 1:1:1 gave the complexes [Cu(bzac)(phen)X]⋅nH_2_O (X: ClO_4_, *n* = 0 (**1**); X: NO_3_, *n* = 1 (**2**)), [Cu(bzac)(bpy)]ClO_4_ (**3**) and [Cu(bzac)(bpy)NO_3_] (**4**) (Figure 1). 

The compound [Cu(bzac)(phen)NO_3_]⋅H_2_O (**2**) was fully characterized as a mononuclear species by single-crystal X-ray diffraction. On the other hand, elemental analysis, IR spectra, and single-crystal X-ray data confirmed the identity of the other complexes with the species reported in the literature [25,26].

All complexes are mononuclear species in which both organic ligands act as a chelate. In all complexes, the oxyanion behaves unidentate, except for complex (**3**), where perchlorate is present as a free ion. As a result, except for complex (**3**), which is an ionic species, the other ones are neutral. The Cu(II) adopts a distorted square pyramidal stereochemistry, except for complex (**3**), where the surrounding is square planar (Figure 1).

We further characterized the compounds by solid-state IR, UV-Vis, EPR spectroscopy, and TGA/DSC MS analysis, and tested their antiproliferative activity.

### 3.1. Description of the Crystal Structure of Complex *(**2**)*

**Figure 2:**
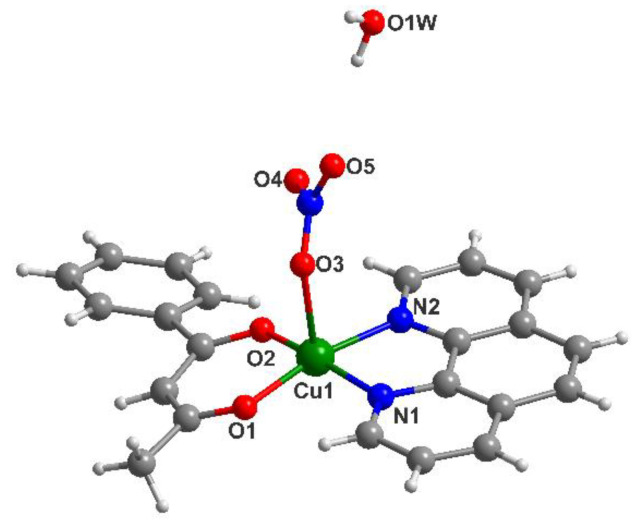
View of the mononuclear unit found in the structure of (**2**).

X-ray diffraction on single crystals for compound (**2**) reveals the presence of a mononuclear unit that crystalizes with one water molecule (Figure 2). A summary of the crystallographic data and the structure refinement are given in Table 1. The [Cu(bzac)(phen)NO_3_] mononuclear species contain the metal ion in a square pyramid surrounding, with the two oxygen atoms from the benzoylacetonato ligand, and the two nitrogen atoms from 1,10-phenanthroline in the basal plane, at distances similar to the ones encountered in other mononuclear units [Cu1-O1 = 1.901(3); Cu1-O2 = 1.909(3); Cu1-N1 = 1.997(4); Cu1-N2 = 2.000(3) Å] [40,41]. The nitrate ligand is coordinated, through its oxygen atom as a terminal ligand, into the apical position [Cu1-O3 = 2.370(4) Å]. Selected bond distances are collected in Table 2. 

**Figure 3:**
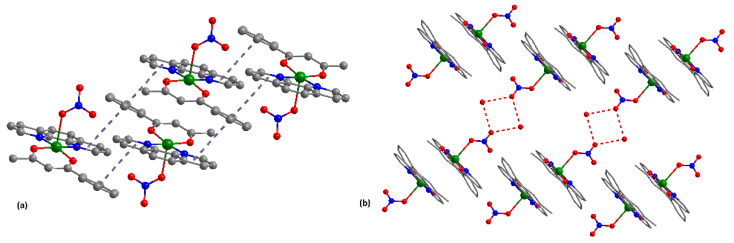
Supramolecular architectures formed through π-π stacking interactions (**a**) and hydrogen bonds (**b**) in (**2**).

Significant π-π stacking interactions occur between the [Cu(bzac)(phen)NO_3_] species, generating a 1D supramolecular architecture (Figure 3a). Each mononuclear unit interacts through the {Cu(bzac)(phen)} fragments with another neighboring unit. The strongest interactions are established between benzoylacetonato ligands and one phenanthroline ligand (centroids distances 3.81 Å).

Interestingly, the neutral species are linked through hydrogen bonds between the water molecule hydrogen and noncoordinated oxygen atom from nitrate anion, [O1w···O5 = 2.968 Å], leading to a supramolecular dimer (Figure 3b). The copper–copper separation within the dimer is Cu···Cu = 12.541 Å.

### 3.2. Physico-Chemical Characterization of Complexes

#### 3.2.1. FT-IR Spectra

The IR spectra of the compounds are very complex in the 400–1700 cm^−1^ range due to the overlapping of the bands of the three ligands, Hbzac, phen/bpy, and ClO_4_/NO_3_ oxyanions.

Compared to the spectra of the two ligands, the spectra of the complexes exhibit multiple bands assigned to overlapping (C=N) and (C=C) stretching vibrations in the 1560–1590 and 1425–1485 cm^−1^ ranges (Table S1). In the spectra of the complexes, the band associated with the delocalized keto-enol bonds appears in the range of 1600 cm^−1^ [42]. The spectrum of (**1**) displays the pattern of unidentate coordination of perchlorate with bands assigned to stretching vibrations *ν*_3_ and *ν*_4_ split in two components and the appearance of a band characteristic for *ν*_1_ vibration at 960 cm^−1^. The perchlorate function of a free ion in compound (**3**) is confirmed by the presence of the characteristic bands at 1090 and 620 cm^−1^, arising from the *ν*_3_ and *ν*_4_ stretching vibrations, respectively [43].

Instead, the spectra of both nitrate species exhibit the pattern characteristic of unidentate behavior. Thus, two components appear in the region characteristic of *ν*_1_ vibration and only one for the other three, but these are shifted compared to the ionic species [43].

The supplementary band at 3475 cm^−1^ in the spectrum of (**2**) was assigned to a hydrated water molecule. In addition, the low-intensity bands in the 430–460 cm^−1^ range were assigned to the (Cu-N) and (Cu-O) stretching vibrations, respectively [42].

#### 3.2.2. UV-Vis Spectra

**Figure 4:**
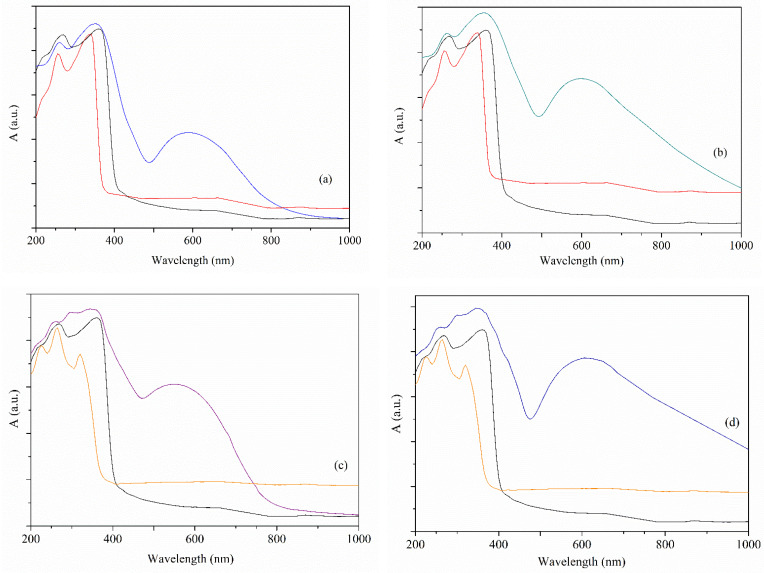
The UV-Vis spectra of (**1**) (blue line, Hbzac-black line, phen-red line) (**a**), (**2**) (blue line) (**b**), (**3**) (purple line, bpy-orange line) (**c**), and (**4**) (blue line) (**d**).

The solid-state UV-Vis spectra of the complexes overlapped with those of the corresponding ligands are presented in Figure 4. The visible region bands are associated with the d-d transitions in all spectra. Hence, the broad band with a maximum at 590, 600, and 605 nm in the spectra of complexes (**1**) (Figure 4a), (**2**) (Figure 4b), and (**4**) (Figure 4d) is assigned to the d_xz_,d_yz_→d_x2−y2_ transition for a square-pyramidal stereochemistry (the values of the τ parameter for copper(II) ion are 1.16, 0.8 and 11.5%, respectively [44]) with a chromophore [Cu(II)N_2_O_3_]. The same transition is responsible for the band at 550 nm in the spectrum of complex (**3**) (Figure 4c), except that it is characteristic of a square-planar stereochemistry with a chromophore [Cu(II)N_2_O_2_] [45].

In addition, the spectra of both organic ligands exhibit two or three bands below 350 nm in the UV region assigned to n→π* and π→π* transitions. In all complexes, these appear as superimposed bands between Hbzac and phen or bpy and are shifted to either lower or higher wavelengths due to coordination.

#### 3.2.3. EPR Spectroscopy

##### Solid State EPR Spectroscopy

**Figure 5:**
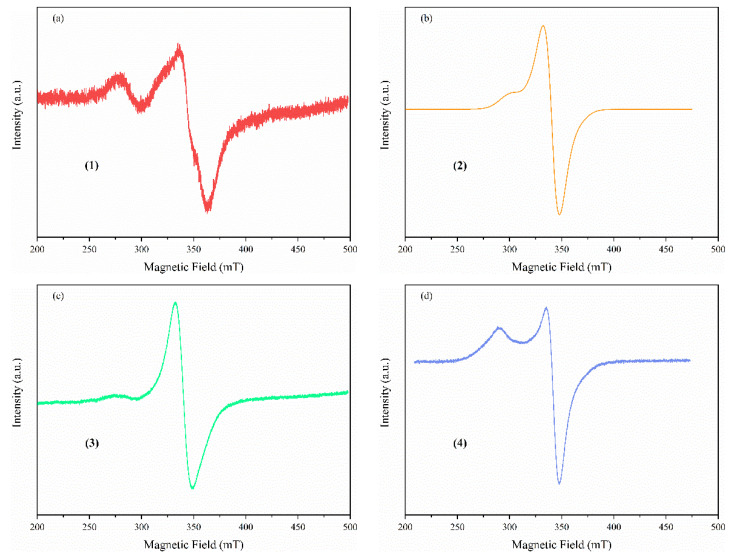
Powder EPR spectra of the complexes ((**1**)-(**a**), (**2**)-(**b**), (**3**)-(**c**), and (**4**)-(**d**)).

The powder Cu(II) complexes were studied using EPR spectroscopy. The measured EPR spectra are presented in Figure 5. All spectra present similar characteristics, with an axial g tensors g_‖_ around 2.44 and g_⊥_ = 2.07 values characteristic of Cu(II) ions either in a square pyramidal (complexes (**1**), (**2**), and (**4**)) or a square-planar stereochemistry (complex (**3**)) as already reported in the literature [46,47,48]. Due to a strong spin–spin exchange interaction, no hyperfine structure is observed at room temperature, which disappears in the relatively broad line width.

##### Solution EPR Spectroscopy

**Figure 6:**
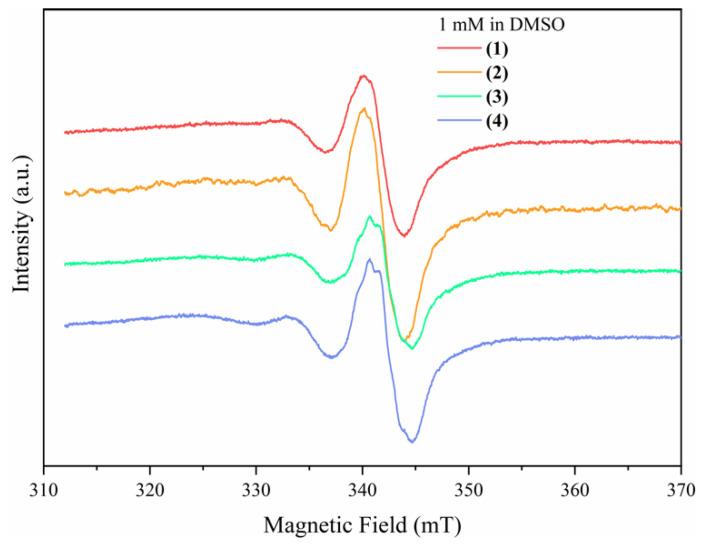
The EPR spectra of the Cu(II)-based complexes in a 1 mM DMSO solution after one week.

The Cu(II) complexes were dissolved in DMSO at a final concentration of 1 mM. Figure 6 shows the EPR spectra of the complexes in solution after one week. All four complexes show excellent stability in solution, which is essential for measurements on cells where the complexes are incubated for two days or longer. All four compounds show relatively broad spectra in solution due to the already mentioned spin–spin exchange interaction between the Cu(II) centers, with an axial g-value of g_‖_ = 2.12 and g_⊥_ = 2.06. Compared with the g-values determined with EPR on the compounds in solid-state, they stay almost unchanged, indicating that the complexes structure is preserved in solution. 

The small shifts in the g-values are due to the changes in the correlation times, which are strongly influenced by the mobility of the molecules. The four normally visible hyperfine lines that should arise from the hyperfine interaction of the Cu(II)-paramagnetic centers with the copper nuclear spin ICu = 3/2 are not observable due to the broadening of the spectra.

#### 3.2.4. Thermal Behavior

The thermal behavior of the complexes was studied using the simultaneous TGA/DSC technique in air coupled with MS analysis to detect the evolved gases. The data are summarized in Table S2.

**Figure 7:**
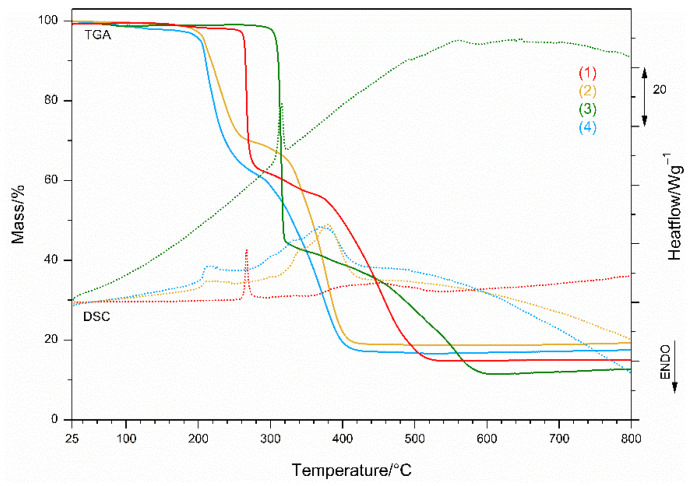
TGA (lines) and DSC (dotted) curves for all complexes.

From the measured curves of all complexes in Figure 7, it is evident that the thermal decomposition process and its pattern are determined by the inorganic ligands, the perchlorate, and the nitrate anion. The decomposition of the complexes containing the perchlorate ion is shifted toward higher temperatures, and the shape of the exothermic peak in the first stage corresponds to an intense and explosive breakdown. The nitrate anion complexes follow a different pattern (Figure S1a,b). The organic ligands are released before the inorganic ions; they decompose exothermically in the last step. The residue of all decomposition pathways in air is an inorganic CuO.

The most thermally stable is complex (**3**), in which perchlorate is present as a free ion that initiates its explosive exothermic decomposition at an onset temperature of 292 °C and ends it in a narrow temperature range at 320 °C. The MS spectra show (Figure S2c, Table S2) the exuberant mass fragmentation characteristic of both organic ligands; moieties from bzac (C_4_H_5_O (*m*/*z* 68), C_6_H_5_ (*m*/*z* 77, 78), (*m*/*z* 105, 85, 43), (*m*/*z* 51, 50), and moieties from bpy (C_5_H_6_N (*m*/*z* 78, 79), (*m*/*z* 51, 52, 50), whose combustion is accompanied by released water (*m*/*z* 17, 18) and carbon dioxide (*m*/*z* 44). In the subsequent exothermic process up to 600 °C, the mass peaks typical of the perchlorate ion Cl (*m*/*z* 35), CHCl (*m*/*z* 49), ClO (*m*/*z* 51, 52), and Cl_2_ (*m*/*z* 70) are strongly pronounced, and also carbon dioxide without water, indicating that the organic matter was burned to carbon black in the first step. The mass peak *m*/*z* 35, present in both decomposition stages, confirms the presence of perchlorate decomposition in the first step, as it is not characteristic of organic ligands.

Complex (**1**), thermally stable up to 125 °C, follows the pattern of perchlorate combustion and rapidly loses its mass up to 292 °C (Figure 7 and Figure S1a). The moiety of the organic ligands bzac and phen (C_6_H_13_N_2_ (*m*/*z* 112, 113, 63, 50)) is slowly released over a further 60 °C (Figure S2a) as *m*/*z* 78, 77, 51, and 50 peaks are formed when exothermic decomposition of the remaining organic matter occurs at 352 °C, releasing water and carbon dioxide as by-products as well as perchlorate decomposition, ending at 569 °C.

The multi-step decomposition of the nitrate complex (**2**) (Figure 7) starts at 155 °C and is accelerated at 214 °C by the decomposition of the organic ligands. Characteristic mass peaks (Table S2, Figure S2b) correspond to the moieties of bzac and phen and the release of nitrogen dioxide (*m*/*z* 30, 46), water, and carbon dioxide. The transformation at 304 °C shows the onset of exothermic decomposition of the nitrate anion and the remaining organic moieties, completed at 509 °C.

However, the nitrate species (**4**) (Figure 7) is the first to undergo exothermic decomposition at 150 °C, with mass peaks (Figure S2d) corresponding to the moieties of bzac, which are further overlaid by peaks of bpy (Table S2). Mass peaks above 304 °C indicate exothermic decomposition of the remaining organic moieties, completed at 538 °C. In all decomposition steps, water and carbon dioxide are released.

### 3.3. Complexes Interaction with Cells and Biological Species

#### 3.3.1. Antiproliferative Activity

**Figure 8:**
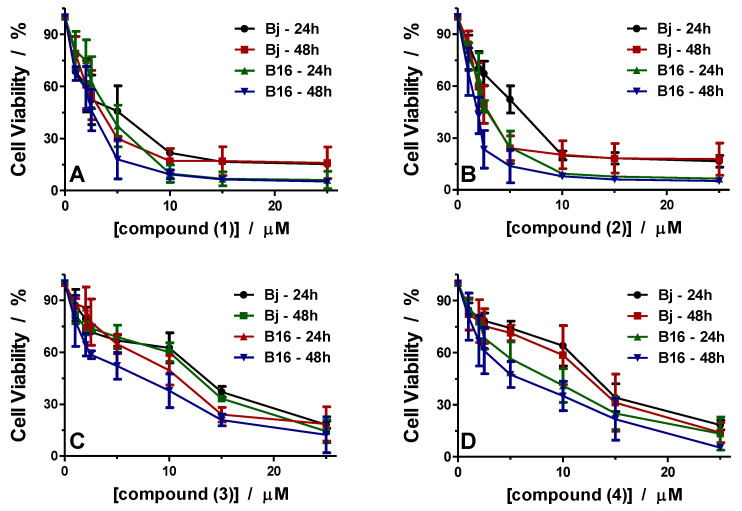
Cell viability of the complexes against BJ and B16 cells after 24 and 48 h for complex (**1**) (**A**), complex (**2**) (**B**), complex (**3**) (**C**), and complex (**4**) (**D**). Data represent the means  ±  SD of at least three independent tests.

Cell viability was evaluated for all compounds at 24 and 48 h against BJ normal skin and murine B16 melanoma cells. The viability curves for all experimental conditions are presented in Figure 8.

The results show that all compounds have slightly enhanced cytotoxicity towards the cancer cell line compared to the normal cell line, but these affect the cells differently. Thus, complexes (**1**) and (**2**) containing 1,10-phenanthroline as a ligand behave similarly (Figure 8A,B). As can be seen, cell viability rapidly decreases to 20% in BJ cells and 10% in B16 cells treated with a concentration of 10 μM of the complexes. Exposure to higher concentrations, up to 25 μM, induces minor changes in cell viability, reaching about 15% for BJ cells and about 5% for B16 cells for both compounds. Furthermore, IC_50_ values were determined for the two compounds, as indicated in Table 3.

For compound (**1**), IC_50_ values are 3.95 μM at 24 h and 2.8 μM at 48 h for BJ cells and 3.48 μM at 24 h and 2.20 μM at 48 h for B16 cells; slightly lower values indicate that compound (**1**) is somewhat more toxic to melanoma cells than to normal skin cells.

This aspect can also be observed from the therapeutic index (TI) calculated and presented in Table 3. The TI was 1.13 after 24 h and 1.27 after 48 h for compound (**1**). Considering the similarities between compounds (**1**) and (**2**), the TI values for compound (**2**) are close to those found for compound (**1**). Values of 4.85 μM at 24 h and 2.48 μM at 48 h were found for BJ cells and 2.65 μM at 24 h and 1.44 μ M at 48 h for B16 cells (Table 3).

Complexes (**1**) and (**2**), like the previously reported 1,10-phenanthroline-containing compounds [16,17,18], showed similar cytotoxicity against melanoma cells in the micromolar range.

Compounds (**3**) and (**4**) containing bpy have a different effect on cell lines BJ and B16 than compounds (**1**) and (**2**). These compounds affect the cells’ viability in a dose-dependent manner, with a slight decrease with increasing concentrations (Figure 8C,D). For both compounds, the viability of B16 cells was more affected than that of BJ cells.

IC_50_ values for compound (**3**) were 10.96 µM at 24 h and 10.43 µM at 48 h when BJ cells were treated, and 8.28 µM at 24 h and 4.65 µM at 48 h for B16 cells (Table 3). The TI values are 1.32 at 24 h and 2.24 at 48 h, indicating that the compounds have more effect against tumor cells with a more extended treatment interval.

For compound (**4**), the IC_50_ values were 11.71 µM at 24 h and 10.33 µM at 48 h for BJ cell treatment and 6.43 µM at 24 h and 4.70 µM at 48 h for B16 cells (Table 3). These values are close to those found for compound (**3**). Calculating the TI values, we found that compound (**4**) also had higher efficiency after 48 h of treatment. TI calculated values were 1.82 after 24 h and 2.19 after 48 h.

Various studies on copper(II) complexes with N-N heterocycle ligands have indicated their potential antitumor activity in the micromolar range [16,18,49,50,51]. Similar results were reported for complexes with both N-N heterocycle and Hbzac, such as (**1**) and (**3**), which were significantly more cytotoxic against MKN-45 cells than 5-fluorouracil after 48 h of incubation [25]. Moreover, Cu(II) species with other β-diketonates proved activity against the chronic myelogenous leukemia cell line [52].

Based on these results, it is evident that Cu(II) complexes with 1,10-phenanthroline are more active than 2,2’-bipyridine complexes, and these results agree with other reported data [16,17,18,49,52,53]. These data have been associated with the enhanced intercalation abilities of this aromatic system.

#### 3.3.2. Effect of Copper(II) Complexes on Cell Cycle Distribution

Considering that the copper(II) complexes can interact with DNA, as mentioned earlier [54], we were interested in verifying whether and how the compounds can interfere with the cell cycle progression. Based on the viability results and the IC_50_ values reported above, we were interested in examining the effect of the compounds at concentrations less than 5 μM values to keep enough cells alive. Cell cycle analysis was performed for tumor cells.

**Figure 9:**
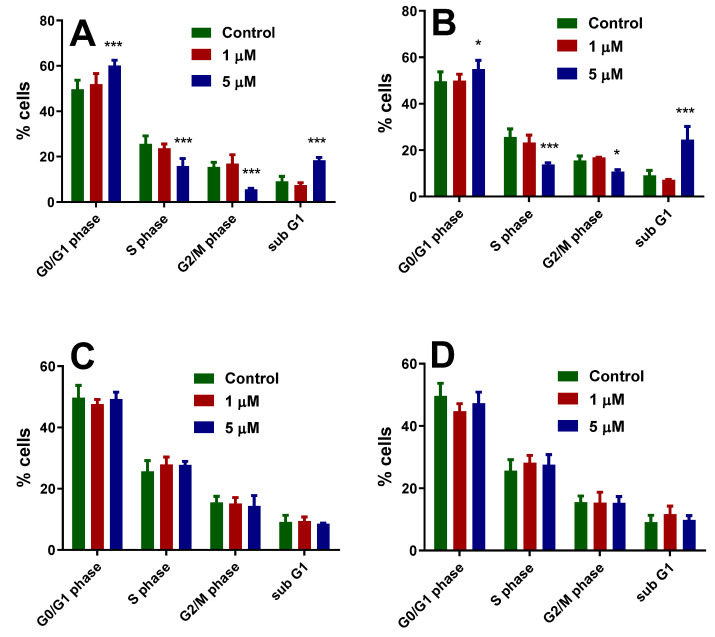
Changes in the cell cycle of B16 cells treated with 1 μM and 5 μM of complexes (**1**) (**A**), (**2**) (**B**), (**3**) (**C**), and (**4**) (**D**) for 24 h. The graphics represent the means  ±  SD of at least three independent tests. * *p*  <  0.05, *** *p* < 0.001 (as compared to negative control using ANOVA one-way followed by Bonferroni’s post-test).

Figure 9 shows the results for B16 cells treated with concentrations of 1 μM and 5 μM of each of the four compounds. The untreated cells, shown as the percentage of cells in each phase, have a distribution of 49.70% in the G0/G1 phase, 25.65% in the S phase, 15.53% in the G2/M phase, and 9.11% in the sub-G1 phase. When treated with compounds (**1**) and (**2**), we see that the cell cycle distribution of B16 cells is affected (Figure 9A,B).

For both compounds, the lower concentration of 1 μM did not significantly alter the cell cycle distribution. However, when B16 cells were treated with 5 μM, we found that both compounds induced cell cycle arrest in the G0/G1 phase and increased the cells in the sub-G1 phase.

Compound (**1**) showed a significant increase (60.14%) of cells in the G0/G1 phase (*p* < 0.001) when treated with 5 μM (Figure 9A). This increase was followed by a significant decrease in the S (15.82%) and G2/M phase (5.59%) (*p* < 0.001) compared to the control cells. In addition, a significant increase (18.43%) in the sub-G1 populations was also observed (*p* < 0.001). The results indicate that compound (**1**) induced G0/G1 cell cycle arrest at a concentration of 5 μM and that the cells also underwent apoptosis.

For compound (**2**), when cells were treated with 5 μM, the percentage of cells that were in the G0/G1 phase significantly increased to 54.90% (*p* < 0.05), while cells in the S and G2/M phase significantly decreased to 13.81% (*p* < 0.001) and 10.78% (*p* < 0.05), respectively. Like compound (**1**), after treating B16 cells with compound (**2**), the percentage of cells that were in the sub-G1 phase significantly increased to 24.49% (*p* < 0.001).

It is known from previous studies that the cell cycle progression is regulated by a series of checkpoints that assess whether a cell can progress with division [46,47]. The two most essential checkpoints are the G1 checkpoint, which allows cells to transition from the G1 to S phase, where DNA synthesis occurs, and the G2 checkpoint, which allows cells to transition from the G2 to M phase, where daughter cell formation by mitosis occurs [55,56].

The G1 checkpoint is activated after detecting DNA damage, among other events, and prevents these cells from transitioning to the S phase. An increase in the number of cells in the sub-G1 phase indicates cells with damaged DNA that have been unable to repair it and have undergone apoptosis. Based on the reported results, we can say that compounds (**1**) and (**2**) can induce DNA fragmentation followed by cell cycle arrest in G0/G1 phase, which eventually leads B16 melanoma cells into apoptosis. Similar findings were previously reported for other copper(II) complexes that caused both G0/G1 cell cycle arrest and apoptosis in various cells [49,54,57,58].

Surprisingly, no changes in the cell cycle were observed for compounds (**3**) and (**4**), or when B16 cells were treated with 1 μM or 5 μM.

#### 3.3.3. Complexes Interaction with ROS

**Figure 10:**
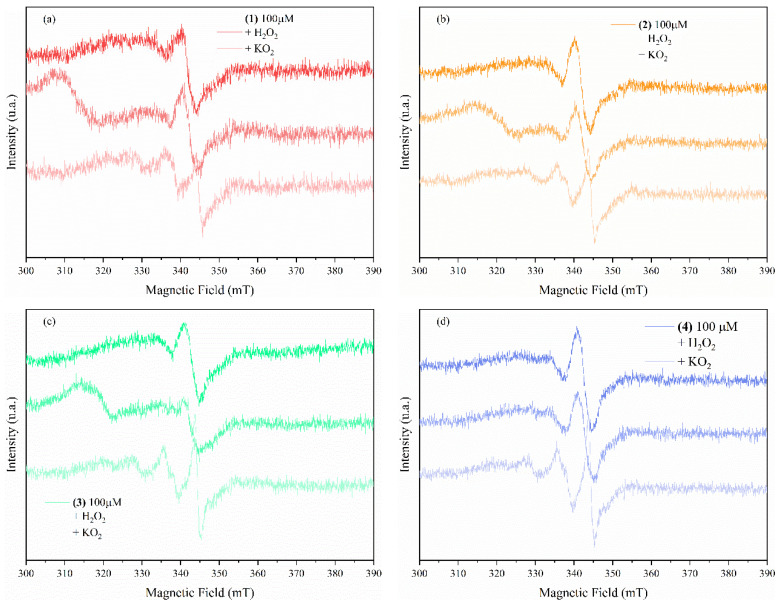
EPR spectra of the complexes ((**1**)-(**a**), (**2**)-(**b**), (**3**)-(**c**), and (**4**)-(**d**)) in a 100 µM DMSO solution and in the presence of KO_2_ and H_2_O_2_.

Figure 10 shows the EPR spectra of the compounds dissolved in DMSO at 100 µM before and after adding KO_2_ and H_2_O_2_ as ROS O_2_⋅^−^ and HO⋅ donors, respectively. The measurements show that compounds (**1**), (**2**), and (**3**) exhibit a scavenging response in the presence of O_2_⋅^−^ radicals by changing the oxidation state of the copper centers, while compound (**4**) shows no changes in its EPR spectrum, indicating that this compound has neither scavenging nor a trapping ability toward superoxide radicals. In contrast, all compounds in the presence of HO⋅ radicals show the trapping ability of these radicals, as indicated by the additional EPR signals in all four EPR spectra.

The Cu(II) complexes (**1**)–(**3**), which exhibit superoxide scavenging abilities, can be reduced inside of the tumor cells by this anion to corresponding Cu(I) species (reaction 1) [59,60]. These Cu(I) complexes can be involved further either in hydrogen peroxide generation (reaction 2) or in its reduction to hydroxyl radical (reaction 3, Fenton-like reaction).

[Cu(II)(N-N)bzac]^+^ + O_2_^−^ → [Cu(I)(N-N)bzac] + O_2_ (1)

[Cu(I)(N-N)bzac] + O_2_^−^ + 2H^+^ → [Cu(II)(N-N)bzac]^+^ + H_2_O_2_ (2)

[Cu(I)(N-N)bzac] + H_2_O_2_ → [Cu(II)(N-N)bzac]^+^ + HOˉ+ HO (3)

On the other hand, the highly reactive hydroxyl radical can induce the DNA cleavage, thus triggering the tumor cell apoptosis.

### 3.4. In Silico Studies

#### 3.4.1. Drug-Likeness, Pharmacokinetics, and Pharmacogenomics Profiles of the Compounds

The results generated from the medicinal chemistry filtering (Lipinski, Ghose, Veber, and Egan) and ADME-Tox analyses are represented in Table 4. Our results show that compounds (**2**)–(**4**) comply with the drug-likeness rules, indicating that these compounds have a possible drug effect and good bioavailability. Even though compound (**1**) has a molecular weight greater than 500 and the Ghose filter does not apply to compound (**1**), we believe that compound (**1**) can also be included in the analysis.

Furthermore, the ADME-predictable properties of the Cu(II)-based complexes were evaluated (Table 5). We evaluated the ADME-Tox characteristics, with emphasis on (i) intestinal absorption, (ii) BBB and CNS permeability, considering that these compounds are planned to be used in antitumor therapies, and (iii) the type of cytochrome for which these compounds are substrates/inhibitors and a renal organic cation transporter 2 (OCT2) substrate. Table 5 also quantifies the predicted toxicity of the complexes. The results revealed that (i) all compounds (**1**)–(**4**) exhibited excellent intestinal absorption, with compounds (**2**) and (**4**) exhibiting outstanding intestinal absorption.

For the BBB permeability considered, we noticed that the complexes recorded good BBB permeability values (log BBB varied from −0.988 to −0.714). All complexes have a reasonable CNS permeability, varying from −3.118 to −1.976. Among the compounds analyzed, compound (**2**) showed suitable permeability for BBB and good permeability in the nervous system. Significant results were recorded for the elimination rate, demonstrating that compounds (**3**) and (**4**) are not renal OCT2 substrates.

In this study, great importance was given to the prediction of the toxicity of the compounds. The results revealed that: (i) none of the compounds (**2**)–(**4**) exhibited AMES toxicity, (ii) compounds (**1**), (**3**), and (**4**) did not induce immunotoxicity, but compounds (**2**)–(**4**) appeared as hepatotoxic species. Very important, the LD_50_ was predicted, the values being in the range 2.968 and 2.672.

Hence, a pharmacogenomic profile of the active compounds was generated (Table 6). The results regarding metabolic pathways showed that Cu(II) complexes are not CYP2D6 substrates/inhibitors, but all compounds are CYP3A4 substrates except CYP3A4 inhibitors (except compound (**4**)). Regarding the affinities of compounds (**1**)–(**4**) to CYP1A2, CYP2C19, or CYP2C9, we mentioned that compound (**2**) inhibits all three cytochromes, but compounds (**3**) and (**4**) have no inhibitory activities of these targets.

#### 3.4.2. Computational Pharmacodynamic Profiles of the Complexes

From the SuperPred database, we extracted the most significant molecular targets for compounds (**1**)–(**4**). We selected the molecular targets with model accuracy greater than 70% and a probability of interaction of at least 90% (Table 7).

We note that the common molecular targets for all compounds were Transcription intermediary factor 1-alpha and Kruppel-like factor 5, whereas Monoamine oxidase A was predicted to be the target for compounds (**1**), (**2**), and (**4**); Tyrosyl-DNA phosphodiesterase 1 was the target for compounds (**1**), (**3**), and (**4**). Literature data mentioned that the Transcription intermediate factor 1-alpha is critically involved in the tumorigenic process [61,62,63]. Additionally, recent studies have mentioned the role and regulation of Kruppel-like factor 5 (KLF5 transcription factor) in cancers [64,65].

From the database SuperPred, the most significant important molecular targets for all complexes were extracted. The molecular targets with more than 70% model accuracy and a probability of interaction not less than 90% were selected (Table 7).

It was noticed that common molecular targets for the compounds were transcription intermediary factor 1-alpha and Kruppel-like factor 5 instead. Monoamine oxidase A was predicted as a target for compounds (**1**), (**2**), and (**4**), while tyrosyl-DNA phosphodiesterase 1 was a target for compounds (**1**), (**3**), and (**4**). Literature data mentioned Transcription intermediary factor 1-alpha is critically involved in the tumorigenic process [61,62,63]. Additionally, recent studies mentioned the roles and regulation of the Kruppel-like factor 5 (KLF5 transcription factor) in cancers [64,65].

## 4. Conclusions

A series of copper perchlorate or nitrate complexes with mixed ligands 1,10-phenanthroline/2,2′-bipyridine and 1-phenyl-1,3-butanedionate were developed as biologically active species. These were characterized as mononuclear, neutral, or ionic species based on single-crystal X-ray diffraction data. The EPR studies evidenced the complexes’ ability to scavenge ROS species, O_2_^−^ and HO. Compounds with 1,10-phenanthroline have the potential to be developed as antitumor agents, considering their efficacy at concentrations around 5 μM and their ability to induce apoptosis in treated cells. Furthermore, compounds (**1**) and (**2**) could be considered in further pharmacological and pharmacokinetic studies due to their lower cytotoxic on the healthy cell line. As more information is needed regarding their efficacy against various other cancers, a new study involving a larger number of healthy and cancerous cell lines can be considered. Bioinformatic tools elucidated the pharmacological profile of the compounds, including important criteria of pharmacokinetic/pharmacodynamics profiles, and important pieces of information about molecular target affinities of compounds were collected. Our results showed that all Cu(II) complexes presented drug-like features, representing an important step when a new compound is proposed as a therapeutic species. Pharmacokinetic properties conclude that studied complexes are not significantly toxic, have a good BBB permeability, and, very importantly, present a good intestinal absorption. Regarding the predicted pharmacodynamic profile of compounds, we concluded that all complexes presented affinities for Transcription intermediary factor 1-alpha, and Kruppel-like factor 5 (KLF5 transcription factor), both critically involved in cancer evolution.

## Figures and Tables

**Table 1:** Crystal data and structure refinement for compound (**2**).

Empirical Formula	C_22_H_19_CuN_3_O_6_
Formula weight	484.94
Temperature/K	293(2)
Crystal system	*triclinic*
Space group	P-1
a/Å	8.7851(7)
b/Å	10.1855(7)
c/Å	12.6653(9)
α/°	74.179(6)
β/°	70.089(7)
γ/°	75.645(6)
Volume/Å^3^	1010.01(14)
Z	2
ρ_calc_g/cm^3^	1.595
μ/mm^−1^	1.128
F(000)	498
Radiation	MoK\a
Reflections collected	9422
Data/restraints/parameters	330/3/296
Goodness-of-fit on F^2^	1.018
Final R indexes [I ≥ 2σ (I)]	0.0702
Final R indexes [all data]	0.0775
Largest diff. peak/hole/e Å^−3^	−1.112, 0.924

**Table 2:** Selected bond lengths (Å) for compound (**2**).

(2)		
Cu1	O1	1.901(3)
Cu1	O2	1.909(3)
Cu1	N1	1.997(4)
Cu1	N2	2.000(3)
Cu1	O3	2.370(4)
N3	O4	1.206(5)
N3	O5	1.251(5)
N1	C1	1.341(5)
N1	C5	1.357(5)
N2	C1	1.314(4)
N1	C2	1.341(3)

**Table 3:** The half-maximal inhibitory concentration (IC_50_) and therapeutic index (TI) of the studied complexes.

Compound	IC_50_ BJ/μM		IC_50_ B16/μM		TI	
	24 h	48 h	24 h	48 h	24 h	48 h
(**1**)	3.95	2.80	3.48	2.20	1.13	1.27
(**2**)	4.85	2.48	2.65	1.44	1.83	1.72
(**3**)	10.96	10.43	8.28	4.65	1.32	2.24
(**4**)	11.71	10.33	6.43	4.70	1.82	2.19

**Table 4:** The drug-likeness features according to Lipinski, Veber, Ghose, and Egan rules.

Compound	Lipinski	Veber	Ghose	Egan	MW g/mol	Log *P_o_*_/*w*_ (XLOGP3)	Bioavailability Score
(**1**)	Yes; one violation: MW > 500	Yes	No; one violation: MW > 480	Yes	505.39	4.77	0.55
(**2**)	Yes	Yes	Yes	Yes	467.94	5.82	0.55
(**3**)	Yes	Yes	No; one violation: MW > 480	Yes	482.37	3.94	0.55
(**4**)	Yes	Yes	Yes	Yes	443.92	5.52	0.55

**Table 5:** Computational pharmacokinetics and toxicity profiles for compounds.

Compound	(1)	(2)	(3)	(4)
HIA	88.33	96.747	84.765	93.026
Log BBB	−0.988	−0.714	−0.94	−0.882
CNS Permeability	−3.09	−2.309	−3.118	−1.976
OCT2 substrate	yes	yes	no	no
AMES toxicity	yes	no	no	no
Immunotoxicity	inactive	active	inactive	inactive
hepatotoxicity	no	yes	yes	yes
Carcinogenicity	inactive	active	inactive	active
LD_50_	2.672	2.606	2.535	2.968

**Table 6:** The inhibitor/substrate features of natural compounds at CYP2D6, CYP3A4, CYP1A2, CYP2C19, and CYP2C9.

Compound	CYP2D6 Substrate/Inhibitor	CYP3A4 Substrate/Inhibitor	CYP1A2 Inhibitor	CYP2C19 Inhibitor	CYP2C9 Inhibitor
(**1**)	no/no	yes/no	yes	no	no
(**2**)	no/no	yes/no	yes	yes	yes
(**3**)	no/no	yes/no	no	no	no
(**4**)	no/no	yes/yes	no	no	no

**Table 7:** The molecular targets of their UNIPROT and PDB code, probability of interactions, and model accuracy of complexes.

**Compound (1)**				
Target Name	UniProt ID	PDB Visualization	Probability (%)	Model accuracy (%)
Monoamine oxidase A	P21397	2Z5Y	95	91
Tyrosyl-DNA phosphodiesterase 1	Q9NUW8	6N0D	95	71
Transcription intermediary factor 1-alpha	O15164	4YBM	92	96
Kruppel-like factor 5	Q13887	Not Available	90	86
**Compound (2)**				
Pregnane X receptor	O75469	6TFI	94	95
Transcription intermediary factor 1-alpha	O15164	4YBM	93	96
Kruppel-like factor 5	Q13887	Not Available	92	86
Monoamine oxidase A	P21397	2Z5Y	91.2	91
**Compound (3)**				
Tyrosyl-DNA phosphodiesterase 1	Q9NUW8	6N0D	96	71
Endoplasmic reticulum-associated amyloid beta-peptide-binding protein	Q99714	2O23	95	70
Transcription intermediary factor 1-alpha	O15164	4YBM	93	96
Nuclear factor NF-kappa-B p105 subunit	P19838	1SVC	93	96
Kruppel-like factor 5	Q13887	Not Available	91	86
**Compound (4)**				
Tyrosyl-DNA phosphodiesterase 1	Q9NUW8	6N0D	96	71
HERG	Q12809	5VA1	96	90
Transcription intermediary factor 1-alpha	O15164	4YBM	96	96
Kruppel-like factor 5	Q13887	Not Available	95	86
Monoamine oxidase A	P21397	2Z5Y	93	91
Cathepsin D	P07339	4OD9	90	99

## Data Availability

Not applicable.

## Supplementary Materials

The following supporting information can be downloaded at: https://www.mdpi.com/article/10.3390/pharmaceutics14081692/s1, Figure S1: TG-DSC graphs in air atmosphere from 25 °C to 600 °C in various combinations: (a) complexes with perchlorate ligand, (b) complexes with nitrate ligand, (c) complexes with bpy ligand, and (d) complexes with phen ligand; Figure S2: TG/DSC–MS graphs of all complexes from 25 to 600 °C in air atmosphere: (a) complex (1), (b) complex (2), (c) complex (3), and, (d) complex (4). Table S1: IR bands (cm−1) together with corresponding assignments; Table S2: Thermal data for complexes in air atmosphere. Representative mass peaks appear on Figure S2.

## References

[1] Manzano C.M., Nakahata D.H., de Paiva R.E.F. (2022). Revisiting metallodrugs for the treatment of skin cancers. Coord. Chem. Rev..

[2] Sander C.S., Hamm F., Elsner P., Thiele J.J. (2003). Oxidative stress in malignant melanoma and non-melanoma skin cancer. Br. J. Dermatol..

[3] Gupta A., Gomes F., Lorigan P. (2017). The role for chemotherapy in the modernmanagement of melanoma. Melanoma Manag..

[4] Denoyer D., Masaldan S., La Fontaine S., Cater M.A. (2015). Targeting copper in cancer therapy: ‘Copper That Cancer’. Metallomics.

[5] Leon I.E., Cadavid-Vargas J.F., Di Virgilio A.L., Etcheverry S.B. (2017). Vanadium, Ruthenium and Copper Compounds: A New Class of Nonplatinum Metallodrugs with Anticancer Activity. Curr. Med. Chem..

[6] Anthony E.J., Bolitho E.M., Bridgewater H.E., Carter O.W.L., Donnelly J.M., Imberti C., Lant E.C., Lermyte F., Needham R.J., Palau M. (2020). Metallodrugs are unique: Opportunities and challenges of discovery and development. Chem. Sci..

[7] Badea M., Uivarosi V., Olar R. (2020). Improvement in the Pharmacological Profile of Copper Biological Active Complexes by Their Incorporation into Organic or Inorganic Matrix. Molecules.

[8] Balsa L.M., Baran E.J., León I.E. (2021). Copper complexes as antitumor agents: In vitro and in vivo evidences. Curr. Med. Chem..

[9] Hasinoff B.B., Yadav A.A., Patel D., Wu X. (2014). The cytotoxicity of the anticancer drug elesclomol is due to oxidative stress indirectly mediated through its complex with Cu(II). J. Inorg. Biochem..

[10] Křikavová R., Vančo J., Trávníček Z., Hutyra J., Dvořák Z. (2016). Design and characterization of highly in vitro antitumor active ternary copper(II) complexes containing 2’-hydroxychalcone ligands. J. Inorg. Biochem..

[11] Mroueh M., Daher C., Hariri E., Demirdjian S., Isber S., Choi E.S., Mirtamizdoust B., Hammud H.H. (2015). Magnetic property, DFT calculation, and biological activity of bis [(μ^2^-chloro) chloro (1,10-phenanthroline)copper (II)] complex. Chem. Biol. Interact..

[12] Hammud H.H., Kortz U., Bhattacharya S., Demirdjian S., Hariri E., Isber S., Sang Choi E., Mirtamizdoust B., Mroueh M., Daher C.F. (2020). Structure, DFT studies, Magnetism and Biological activity of Bis[(μ-azido)-chloro-(1,10-phenanthroline)-copper(II)] complex. Inorg. Chim. Acta..

[13] Slator C., Molphy Z., McKee V., Long C., Brown T., Kellett A. (2018). Di-copper metallodrugs promote NCI-60 chemotherapy via singlet oxygen and superoxide production with tandem TA/TA and AT/AT oligonucleotide discrimination. Nucleic Acids Res..

[14] Ahmad M., Suhaimi S.-N., Chu T.-L., Abdul Aziz N., Mohd Kornain N.-K., Samiulla D.S., Lo K.-W., Ng C.-H., Khoo A.S.-B. (2018). Ternary copper(II) complex: NCI60 screening, toxicity studies, and evaluation of efficacy in xenograft models of nasopharyngeal carcinoma. PLoS ONE.

[15] Nakahata D.H., de Paiva R.E.F., Lustri W.R., Ribeiro C.M., Pavan F.R., da Silva G.G., Ruiz A.L.T.G., de Carvalho J.E., Corbi P.P. (2018). Sulfonamide-containing copper(II) metallonucleases: Correlations with in vitro antimycobacterial and antiproliferative activities. J. Inorg. Biochem..

[16] Olar R., Badea M., Bacalum M., Raileanu M., Ruta L.L., Farcasanu I.C., Rostas A.M., Vlaicu I.D., Popa M., Chifiriuc M.C. (2021). Antiproliferative and antibacterial properties of biocompatible copper(II) complexes bearing chelating N,N-heterocycle ligands and potential mechanisms of action. Biometals.

[17] Rostas A.M., Badea M., Ruță L.L., Farcașanu I.C., Maxim C., Chifiriuc M.C., Popa M., Luca M., Celan Korošin N., Cerč Korošec R. (2020). Copper(II) complexes with mixed heterocycle ligands as promising antibacterial and antitumor species. Molecules.

[18] Ruta L.L., Farcasanu I.C., Bacalum M., Raileanu M., Rostas A.M., Daniliuc C.G., Chifiriuc M.C., Marutescu L., Popa M., Badea M. (2021). Biological activity of triazolopyrimidine copper(II) complexes modulated by an auxiliary N-N-chelating heterocycle ligands. Molecules.

[19] Ruan B.-F., Liang Y.-K., Liu W.-D., Wu J.-Y., Tian Y.-P. (2012). Synthesis, characterization, and antitumor activities of two copper(II) complexes with pyrazole derivatives. J. Coord. Chem..

[20] Borges L.J.H., Bull É.S., Fernandes C., Horn A., Azeredo N.F., Resende J.A.L.C., Freitas W.R., Carvalho E.C.Q., Lemos L.S., Jerdy H. (2016). In vitro and in vivo studies of the antineoplastic activity of copper (II) compounds against human leukemia THP-1 and murine melanoma B16-F10 cell lines. Eur. J. Med. Chem..

[21] Kalinowska-Lis U., Szabłowska-Gadomska I., Lisowska K., Ochocki J., Małecki M., Felczak A. (2017). Cytotoxic and Antimicrobial Properties of Copper (II) Complexes of Pyridine and Benzimidazole Derivatives. Z. Anorg. Allg. Chem..

[22] Mariani D., Ghasemishahrestani Z., Freitas W., Pezzuto P., Costa-da-Silva A.C., Tanuri A., Kanashiro M.M., Fernandes C., Horn A., Pereira M.D. (2021). Antitumoral synergism between a copper(II) complex and cisplatin improves in vitro and in vivo anticancer activity against melanoma, lung and breast cancer cells. Biochim. Biophys. Acta.

[23] Gurudevaru C., Gopalakrishnan M., Senthilkumar K., Hemachandran H., Siva R., Srinivasan T., Velmurugan D., Shanmugan S., Palanisami N. (2017). Synthesis and structural and DNA binding studies of mono and dinuclear copper (II) complexes constructed with –O and –N donor ligands: Potential anti-skin cancer drugs. Appl. Organometal. Chem..

[24] Monti E., Paracchini L., Piccinini F., Malatesta V., Morazzoni F., Supino R. (1990). Cardiotoxicity and antitumor activity of a copper (II)-doxorubicin chelate. Cancer Chemother. Pharmacol..

[25] Eshaghi Malekshah R., Salehi M., Kubicki M., Khaleghian A. (2018). New mononuclear copper(II) complexes from β-diketone and β-keto ester N-donor heterocyclic ligands: Structure, bioactivity and molecular simulation studies. J. Coord. Chem..

[26] Zhang L., Xu D., Xu Y., Gu J. (1997). (Benzoylacetonato-*O*,*O*’)(2,2’-bipyridine-*N*,*N*’)(nitrato-*O*)copper(II). Acta Cryst..

[27] Avram S., Mernea M., Bagci E., Hritcu L., Borcan L.C., Mihailescu D.F. (2022). Advanced Structure-activity Relationships Applied to Mentha spicata L. Subsp. spicata Essential Oil Compounds as AChE and NMDA Ligands, in Comparison with Donepezil, Galantamine and Memantine—New Approach in Brain Disorders Pharmacology. CNS Neurol. Disord..

[28] Udrea A.M., Puia A., Shaposhnikov S., Avram S. (2018). Computational approaches of new perspectives in the treatment of depression during pregnancy. Farmacia.

[29] Avram S., Bologa C., Flonta M.L. (2005). Quantitative structure-activity relationship by CoMFA for cyclic urea and nonpeptidecyclic cyanoguanidine derivatives on the wild type and mutants HIV-1 protease. J. Mol. Model..

[30] Sheldrick G.M. (1998). SHELXL-97, Program for Crystal Structure Refinement.

[31] Bordei Telehoiu A.T., Nuță D.C., Căproiu M.T., Dumitrascu F., Zarafu I., Ioniță P., Bădiceanu C.D., Avram S., Chifiriuc M.C., Bleotu C. (2020). Design, Synthesis and In Vitro Characterization of Novel Antimicrobial Agents Based on 6-Chloro-9H-carbazol Derivatives and 1,3,4-Oxadiazole Scaffolds. Molecules.

[32] Lipinski C.A., Lombardo F., Dominy B.W., Feeney P.J. (2001). Experimental and Computational Approaches to Estimate Solubility and Permeability in Drug Discovery and Development Settings 1PII of Original Article: S0169-409X9600423-1. Adv. Drug Deliv. Rev..

[33] Ghose A.K., Viswanathan V.N., Wendoloski J.J. (1999). A Knowledge-Based Approach in Designing Combinatorial or Medicinal Chemistry Libraries for Drug Discovery. 1. A Qualitative and Quantitative Characterization of Known Drug Databases. J. Comb. Chem..

[34] Veber D.F., Johnson S.R., Cheng H.-Y., Smith B.R., Ward K.W., Kopple K.D. (2002). Molecular Properties That Influence the Oral Bioavailability of Drug Candidates. J. Med. Chem..

[35] Egan W.J., Merz K.M., Baldwin J.J. (2000). Prediction of Drug Absorption Using Multivariate Statistics. J. Med. Chem..

[36] Daina A., Michielin O., Zoete V. (2017). SwissADME: A Free Web Tool to Evaluate Pharmacokinetics, Drug-Likeness and Medicinal Chemistry Friendliness of Small Molecules. Sci. Rep..

[37] http://biosig.unimelb.edu.au/pkcsm/.

[38] https://prediction.charite.de/.

[39] https://prediction.charite.de/subpages/target_result.php.

[40] Madalan A.M., Avarvari N., Andruh M. (2006). Metal complexes as second-sphere ligands. New J. Chem..

[41] Dumitru I., Ene C.D., Ofiteru A.M., Paraschivescu C., Madalan A.M., Baciu I., Farcasanu I.C. (2012). Identification of [CuCl(acac)(tmed)], a copper(II) complex with mixed ligands, as a modulator of Cu, Zn superoxide dismutase (Sod1p) activity in yeast. J. Biol. Inorg. Chem..

[42] Nakamoto K. (2009). Infrared and Raman Spectra of Inorganic and Coordination Compounds, Part B, Applications in Coordination, Organometallic, and Bioinorganic Chemistry.

[43] Hathaway B.J., Wilkinson G., Gillard R.D., McCleverty J.A. (1987). Oxyanions. Comprehensive Coordination Chemistry.

[44] Addison A.W., Rao T.N., Reedijk J., van Rijn J., Verschor G.C. (1984). Synthesis, structure, and spectroscopic properties of copper(II) compounds containing nitrogen–sulphur donor ligands; the crystal and molecular structure of aqua[1,7-bis(N-methylbenzimidazol-2′-yl)-2,6-dithiaheptane]copper(II) perchlorate. J. Chem. Soc. Dalton Trans..

[45] Lever A.B.P. (1986). Inorganic Electronic Spectroscopy.

[46] Anatole A., Bleaney B. (2012). Electron Paramagnetic Resonance of Transition Ion.

[47] Maxim C., Badea M., Rostas A.M., Chifiriuc M.C., Pircalabioru G.G., Avram S., Olar R. (2022). Copper (II) species with 1-(o-tolyl) biguanide: Structural characterization, ROS scavenging, antibacterial activity, biocompatibility and in silico studies. Appl. Organomet. Chem..

[48] Garriba E., Micera G. (2006). The Determination of the Geometry of Cu(II) Complexes An EPR Spectroscopy Experiment. J. Chem Ed..

[49] Machado P.H.A., Paixão D.A., Campos Lino R., de Souza T.R., de Souza Bontempo N.J., Sousa L.M., Van Petten de Vasconcelos Azevedo F., Capelari Orsolin P., Alves Pereira Lima P.M., Castro Martins I. (2021). A selective Cu^II^ complex with 4-fluorophenoxyacetic acid hydrazide and phenanthroline displays DNA-cleaving and pro-apoptotic properties in cancer cells. Sci. Rep..

[50] Levín P., Ruiz M.C., Romo A.I.B., Nascimento O.R., Di Virgilio A.L., Oliver A.G., Ayala A.P., Diógenes I.C.N., León I.E., Lemus L. (2021). Water-mediated reduction of [Cu(dmp)_2_(CH_3_CN)]^2+^: Implications of the structure of a classical complex on its activity as an anticancer drug. Inorg. Chem. Front..

[51] Ruiz M.C., Perelmulter K., Levín P., Romo A.I.B., Lemus L., Bollati -Fogolín M., León I.E., Di Virgilio A.L. (2022). Antiproliferative activity of two copper (II) complexes on colorectal cancer cell models: Impact on ROS production, apoptosis induction and NF-κB inhibition. Eur. J. Pharm. Sci..

[52] Almeida J.C., Paixão D.A., Marzano I.M., Ellena J., Pivatto M., Lopes N.P., Ferreira A.M.D.C., Pereira-Maia E.C., Guilardi S., Guerra W. (2015). Copper(II) complexes with β-diketones and N-donor heterocyclic ligands: Crystal structure, spectral properties, and cytotoxic activity. Polyhedron.

[53] Burgos-López Y., Balsa L.M., Piro O.E., León I.E., García-Tojal J., Echeverría G.A., González-Baró A.C., Parajón-Costa B.S. (2022). Tridentate acylhydrazone copper(II) complexes with heterocyclic bases as coligands. Synthesis, spectroscopic studies, crystal structure and cytotoxicity assays. Polyhedron.

[54] Elsayed S.A., Elnabky I.M., di Biase A., El-Hendawy A.M. (2022). New mixed ligand copper(II) hydrazone-based complexes: Synthesis, characterization, crystal structure, DNA/RNA/BSA binding, in vitro anticancer, apoptotic activity, and cell cycle analysis. Appl. Organomet. Chem..

[55] Barnum K.J., O’Connell M.J. (2014). Cell Cycle Regulation by Checkpoints. Methods Mol. Biol..

[56] Shaltiel I.A., Krenning L., Bruinsma W., Medema R.H. (2015). The same, only different—DNA damage checkpoints and their reversal throughout the cell cycle. J. Cell Sci..

[57] Hajrezaie M., Paydar M., Zorofchian Moghadamtousi S., Hassandarvish P., Gwaram N.S., Zahedifard M., Rouhollahi E., Karimian H., Looi C.Y., Ali H.M. (2014). A Schiff Base-Derived Copper (II) Complex Is a Potent Inducer of Apoptosis in Colon Cancer Cells by Activating the Intrinsic Pathway. Sci. World J..

[58] Gu Y.-Q., Zhong Y.-J., Hu M.-Q., Li H.-Q., Yang K., Dong Q., Liang H., Chen Z.-F. (2022). Terpyridine copper(II) complexes as potential anticancer agents by inhibiting cell proliferation, blocking the cell cycle and inducing apoptosis in BEL-7402 cells. Dalton Trans..

[59] Wehbe M., Leung A.W.Y., Abrams M.J., Orvig C., Bally M.B. (2017). A Perspective—Can copper complexes be developed as a novel class of therapeutics?. Dalton Trans..

[60] Esmaeili L., Perez M.G., Jafari M., Paquin J., Ispas-Szabo P., Pop V., Andruh M., Byers J., Mateescu M.A. (2019). Copper complexes for biomedical applications: Structural insights, antioxidant activity and neuron compatibility. J. Inorg. Biochem..

[61] Zhang L.H., Yin Y.H., Chen H.Z., Feng S.Y., Liu J.L., Chen L., Fu W.L., Sun G.C., Yu X.G., Xu D.G. (2020). TRIM24 Promotes Stemness and Invasiveness of Glioblastoma Cells via Activating SOX2 Expression. Neuro-Oncol..

[62] Yu T., Gan S., Zhu Q., Dai D., Li N., Wang H., Chen X., Hou D., Wang Y., Pan Q. (2019). Modulation of M2 macrophage polarization by the crosstalk between Stat6 and Trim24. Nat. Commun..

[63] Klapper L., Idel C., Kuppler P., Jagomast T., von Bernuth A., Bruchhage K.L., Rades D., Offermann A., Kirfel J., Perner S. (2022). TRIM24 Expression as an Independent Biomarker for Prognosis and Tumor Recurrence in HNSCC. J. Pers. Med..

[64] Luo Y., Chen C. (2021). The roles and regulation of the KLF5 transcription factor in cancers. Cancer Sci..

[65] Kim C.K., Saxena M., Maharjan K., Song J.J., Shroyer K.R., Bialkowska A.B., Shivdasani R.A., Yang V.W. (2020). Krüppel-like Factor 5 Regulates Stemness, Lineage Specification, and Regeneration of Intestinal Epithelial Stem Cells. Cell. Mol. Gastroenterol. Hepatol..

