# Supplementary material for: Antiproliferative Copper(II) Complexes Bearing Mixed Chelating Ligands: Structural Characterization, ROS Scavenging, In Silico Studies, and Anti-Melanoma Activity
Source: Pharmaceutics. 2022 Aug 14;14(8):1692. doi: 10.3390/pharmaceutics14081692 (PMC9416163; doi:10.3390/pharmaceutics14081692)
Supplement: Supplementary file 1 [file pharmaceutics-14-01692-s001.zip › pharmaceutics-1832578-supplementary.pdf]

pharmaceutics

MDPI

Antiproliferative copper(II) complexes bearing mixed chelating ligands: structural characterization, ROS scavenging, antitumor activity and *in silico* studies

Rodica Olar [1,*], Catalin Maxim [1], Mihaela Badea [1], Mihaela Bacalum [2], Mina Raileanu [2,3], Speranța Avram [4*], Natasa Celan Korosin [5], Teodora Burlanescu [6] and Arpad Mihai Rostas [7]

1 University of Bucharest, Faculty of Chemistry, Department of Inorganic Chemistry, 90-92 Panduri Str., 050663 Bucharest, Romania
2 Department of Life and Environmental Physics, Horia Hulubei National Institute for Physics and Nuclear Engineering, 30 Reactorului Str., 077125 Magurele-Ilfov, Romania
3 University of Bucharest, Faculty of Physics, Department of Electricity, Solid State and Biophysics, 405A Atomiștilor Str., 077125 Magurele-Ilfov, Romania
4 University of Bucharest, Faculty of Biology, Department of Anatomy, Animal Physiology and Biophysics, 91-95, Splaiul Independenței, Bucharest, Romania
5 University of Ljubljana, Faculty of Chemistry and Chemical Technology, Večna pot 113, 1000 Ljubljana, Slovenia
6 National Institute of Materials Physics, Laboratory of Optical Processes in Nanostructure Materials, 405A Atomiștilor Str., 077125 Magurele-Ilfov, Romania
7 National Institute of Materials Physics, Laboratory of Atomic Structures and Defects in Advanced Materials, LASDAM, 405A Atomiștilor Str., 077125 Magurele-Ilfov, Romania
* Correspondence: rodica.olar@chimie.unibuc.ro (R.O.); speranta.avram@bio.unibuc.ro (S.A.)

Table S1. IR bands (cm^{-1}) together with corresponding assignments.

(1)	(2)	(3)	(4)	Assignments
-	3475m	-	-	ν(H_2O)
1615 m	1615 m	1605 m	1605 m	ν(C=O)
1590 m	1590 m	1590 m	1590 m	ν(C=N)
1560 s	1560 s	1560 m	1560 s	
-	1520 s<br>1320 m	-	1520 s<br>1315 s	$ν_3(NO_3)$
1485 m	1485 m	1485 m	1485 m	ν(C=C)
1450 m	1450 m	1450 m	1450 m	
1425 m	1425 m	1435 m	1435 m	
1105 vs<br>1065 s	1020 m	1090 vs	1025 m	$ν_3(ClO_4)$/ $ν_1(NO_3)$
960 m	-	-	-	$ν_1(ClO_4)$
-	850 m	-	850 m	$ν_2(NO_3)$
-	715 m	-	715 m	$ν_4(NO_3)$
650 w<br>620 m	-	620 m	-	$ν_4(ClO_4)$
445 w	445 w	450 w	460 w	ν(Cu-N)
430 w	430 w	440 w	435 w	ν(Cu-O)

s-strong; m-medium, w-weak.

Table S2. Thermal data for complexes in air atmosphere. Representative mass peaks appear on Supplementary Figure S2.

Compound	Step	Thermal effect	Temperature range / °C	Δm_{exp} /%	Identified product by EGA with corresponding *m*/*z* values
(1)	1.	Exothermic	125-292	37.75	moieties from bzac (C_6H_5 (77, 78), 105, (51, 50), moieties from phen ($C_6H_{13}N_2$ (112, 113), 63, 50), perchlorate (Cl (35), CHCl (49), ClO (51, 52), H_2O (17, 18), CO_2 (44)
	2.	Exothermic	292-352	5.07	moieties from bzac and phen (77, 78, 51, 50), H_2O (17, 18), CO_2 (44)
	3.	Exothermic	352-569	41.77	perchlorate (Cl (35), CHCl (49), ClO (50, 51), Cl_2 (70)), H_2O (17, 18), CO_2 (44), still some organic moiety
(2)	1.	Endothermic	25-155	2.96	H_2O (17, 18)
	2.	Exothermic	155-214	7.73	moieties from bzac (C_4H_5O (68), C_6H_5 (77, 78), 51), H_2O (17, 18), CO_2 (44)
	3.	Exothermic	214-304	23.99	moieties from bzac (C_4H_5O (68), C_6H_5 (77, 78), 51), moieties from phen ($C_6H_{13}N_2$ (112, 113), 63, 50), nitrate (NO (30), NO_2 (46)), H_2O (17, 18), CO_2 (44)
	4.	Exothermic	304-509	48.65	moieties from bzac (C_4H_5O (68), C_6H_5 (77, 78), moieties from phen ($C_6H_{13}N_2$ (112, 113), 63, 50), nitrate (NO (30), NO_2 (46)), H_2O (17, 18), CO_2 (44)
(3)	1.	Exothermic	251-320	54.36	moieties from bzac (C_4H_5O (68), C_6H_5 (77, 78), 105, 85, 43, (51, 50), moieties from bpy (C_5H_6N (78, 79), (51, 52, 50), perchlorate (Cl (35), CHCl (49), ClO (51, 52)), H_2O (17, 18), CO_2 (44)
	2.	Exothermic	320-600	31.65	perchlorate (Cl (35), CHCl (49), ClO (51, 52), Cl_2 (70)), CO_2 (44)
(4)	1.	Exothermic	150-278	37.75	moieties from bzac (C_4H_5O (68, 69), C_6H_5 (77, 78), 60, 51), nitrate (NO (30), NO_2 (46)), H_2O (17, 18), CO_2 (44)
	2.	Exothermic	278-304	4.70	moieties from bzac (C_6H_5 (77, 78), 105, 51, 43) moieties from bpy (C_5H_6N (78, 79), (51, 52, 50), nitrate (NO (30), NO_2 (46)), H_2O (17, 18), CO_2 (44)
	3.	Exothermic	304-337	11.29	still some organic moiety, nitrate (NO (30), NO_2 (46)), H_2O (17, 18), CO_2 (44)
	4	Exothermic	337-538	30.03	nitrate (NO (30), NO_2 (46)), H_2O (17, 18), CO_2 (44)

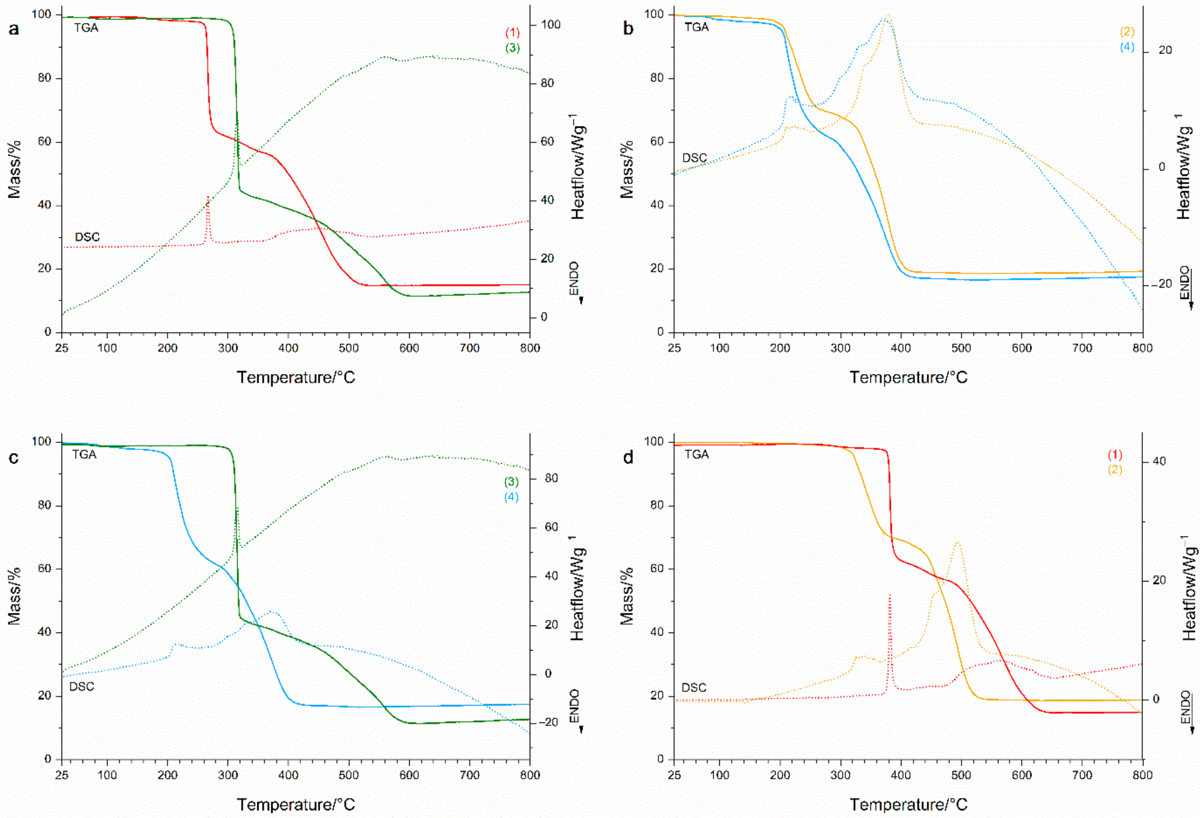

Figure S1. TG-DSC graphs in air atmosphere from 25 °C to 600 °C in various combinations: **a)** complexes with perchlorate ligand, **b)** complexes with nitrate ligand, **c)** complexes with bpy ligand, and **d)** complexes with phen ligand.

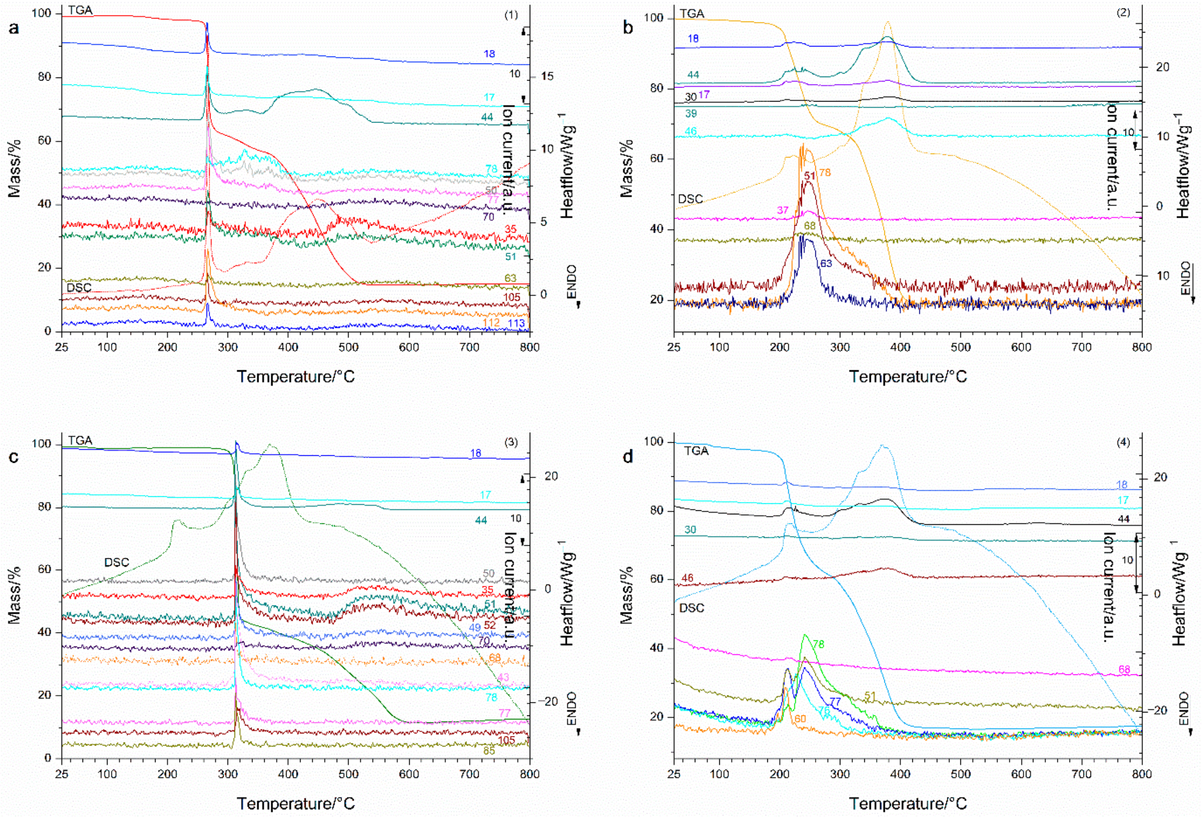

Figure S2. TG/DSC–MS graphs of all complexes from 25 to 600 °C in air atmosphere: **a)** complex (**1**), **b)** complex (**2**), **c)** complex (**3**), and, **d)** complex (**4**).
